# Elucidating the role of highly homologous *Nicotiana benthamiana* ubiquitin E2 gene family members in plant immunity through an improved virus-induced gene silencing approach

**DOI:** 10.1186/s13007-017-0210-6

**Published:** 2017-07-21

**Authors:** Bangjun Zhou, Lirong Zeng

**Affiliations:** 1 Center for Plant Science Innovation, Department of Plant Pathology, University of Nebraska, Lincoln, NE 68583 USA; 2 Southern Regional Collaborative Innovation Center for Grain and Oil Crops, Hunan Agricultural University, Changsha, 410128 China

**Keywords:** Virus-induced gene silencing, Off-target gene, Highly homologous gene family, *Tobacco rattle virus*, Ubiquitin-conjugating enzyme, Plant immunity

## Abstract

**Background:**

Virus-induced gene silencing (VIGS) has been used in many plant species as an attractive post transcriptional gene silencing (PTGS) method for studying gene function either individually or at large-scale in a high-throughput manner. However, the specificity and efficiency for knocking down members of a highly homologous gene family have remained to date a significant challenge in VIGS due to silencing of off-targets.

**Results:**

Here we present an improved method for the selection and evaluation of gene fragments used for VIGS to specifically and efficiently knock down members of a highly homologous gene family. Using this method, we knocked down twelve and four members, respectively of group III of the gene family encoding ubiquitin-conjugating enzymes (E2) in *Nicotiana benthamiana*. Assays using these VIGS-treated plants revealed that the group III E2s are essential for plant development, plant immunity-associated reactive oxygen species (ROS) production, expression of the gene *NbRbohB* that is required for ROS production, and suppression of immunity-associated programmed cell death (PCD) by AvrPtoB, an effector protein of the bacterial pathogen *Pseudomons syringae*. Moreover, functional redundancy for plant development and ROS production was found to exist among members of group III E2s.

**Conclusions:**

We have found that employment of a gene fragment as short as approximately 70 base pairs (bp) that contains at least three mismatched nucleotides to other genes within any 21-bp sequences prevents silencing of off-target(s) in VIGS. This improved approach in the selection and evaluation of gene fragments allows for specific and efficient knocking down of highly homologous members of a gene family. Using this approach, we implicated *N. benthamiana* group III E2s in plant development, immunity-associated ROS production, and suppression of multiple immunity-associated PCD by AvrPtoB. We also unraveled functional redundancy among group III members in their requirement for plant development and plant immunity-associated ROS production.

**Electronic supplementary material:**

The online version of this article (doi:10.1186/s13007-017-0210-6) contains supplementary material, which is available to authorized users.

## Background

Functional genomics makes use of the wealth of data produced by genome sequencing and transcriptomic projects and entails the development and application of global scale approaches to study gene function. In the past decade, we have witnessed remarkable advancements in technologies that have revolutionized many high-throughput research methods in unraveling gene function at genome scale. Various tools for knocking out or knocking down genes of interest have been developed. Examples of such tools include the clustered regularly interspaced short palindromic repeats (CRISPR)/CRISPR-associated9 (Cas9) system [1–3], silencing of genes by RNA interference (RNAi) [4], and mutation of genes by transposable element [5], *Agrobacterium*-mediated T-DNA insertion [6, 7], or chemicals, such as ethyl methanesulfonate [8] and targeting induced local lesions in genomes (TILLING) [9]. Among these tools, silencing of genes via RNAi has largely been used in reverse genetics and involves direct alteration in the expression of a gene and subsequent identification of gene function based on the phenotypes that result from such change in gene expression.

RNAi functions via a sequence-specific RNA degradation mechanism that is triggered by double stranded RNA (dsRNA) and has been shown to be the primary mechanism underlying anti-viral infection by plants [10–13]. By using recombinant virus carrying a partial sequence from a host gene, the RNAi-mediated antiviral mechanism can be utilized to silence the corresponding host gene, a technique that has been defined as virus-induced gene silencing (VIGS) [14, 15]. During VIGS, as the recombinant virus spreads out systemically in the plant, dsRNAs corresponding to the host gene are produced and then cleaved into small interference RNAs (siRNAs) in the length of 21–24 nucleotides (nt). These siRNAs are ultimately incorporated into the RNA-induced silencing complex (RISC) to degrade the target mRNAs [16]. Since the first report in 1995 [17], VIGS has been widely used as an attractive post transcriptional gene silencing (PTGS) method for studying the function of genes individually or in a high-throughput manner. Different viruses have been engineered for VIGS in various plant species. To date, VIGS systems have been established for tomato [15], tobacco [18], Arabidopsis [19], barley [20], *Medicago truncatula* [21], soybean [22], cotton [23], grasses [24], strawberry [25], and pepper [26]. Besides being used in the whole plant, VIGS has also been employed to study gene function in specific plant organs, such as fruit [25, 27] and root [28]. It is now well accepted that VIGS is a simple, quick and cost-effective method for high throughput study of gene function.

The *Tobacco mosaic virus* (TMV)- and *Tobacco rattle virus* (TRV)-based vectors for VIGS were the first to be engineered for targeted silencing of a plant gene [15, 29–31]. The TRV infects many plant species and TRV-based VIGS can probably be applied to the widest known host range among any engineered plant viruses for VIGS [32]. TRV has a positive sense single-stranded RNA genome consisting of two components, referred to as RNA1 and RNA2. RNA1 encodes genes for viral replication and movement, whereas RNA2 encodes the coat protein and some nonessential structural proteins that can be deleted to allow the insertion of foreign sequence fragment [15]. Gene fragments in the length of 200–1500 bp have been used successfully to induce TRV-based VIGS [30, 33]. Like other plant viruses engineered for VIGS, a major concern of TRV-based VIGS is the silencing of off-targets due to possible sequence homology between the gene-of-interest and the off-target genes. Efforts to minimize such non-specific gene silencing have been attempted. For example, the online software algorithm called SGN VIGS tool has been developed to facilitate the identification of unique gene fragment(s) for building VIGS constructs [34]. However, the tool allows selection of gene fragments that are equal to or are more than 100 bp only, which limits the chances of identifying suitable sequence regions to be used for specific silencing in VIGS.

Despite the concern on silencing of off-targets, the VIGS tool has been widely used in the study of plant immunity in the past decade. Many key components of the plant immune system have been identified with the help of the VIGS tool. It is known now, during co-evolution with microbes, plants have developed a multi-layered defense strategy to protect against pathogens. At the front line of the strategy, plants initiate pattern-triggered immunity (PTI) upon detection of microbe-associated and damage-associated molecular patterns (MAMPs and DAMPs) through the cell surface-anchored pattern-recognition receptors (PRRs). PTI is intimately associated with rapid calcium influx, production of reactive oxygen species (ROS), induction of mitogen activated protein kinase (MAPK) cascades, defense gene expression, and callose deposition and is effective to ward off a broad range of microbes [35]. However, pathogens have evolved to overcome PTI by deploying during the infection process various effector proteins into the host cells [36, 37]. In response, plants have evolved intracellular nucleotide-binding leucine-rich repeat (NB-LRR) receptors (also known as resistance proteins) that either directly or indirectly monitor the activity of effector proteins to initiate effector-triggered immunity (ETI), the second line of plant defense strategy. Many PTI-associated plant responses, such as accumulation of calcium and ROS and induction of MAPK cascades are also accompanied with ETI. Importantly, ETI is also often associated with a rapid induction of programmed cell death (PCD) called hypersensitive response (HR) at the site of infection, which serves to limit the spread of the pathogen, particularly biotrophic pathogens.

Using tomato genes encoding the ubiquitin-conjugating enzymes (E2) as the query, we recently identified 40 *Nicotiana benthamiana* ubiquitin E2 genes by BLAST [38, 39]. The forty *N. benthamiana* E2 genes were classified into thirteen groups (Additional file 1: Fig. S1) and the twelve members of group III are highly homologous. In the present work, we demonstrate an improved VIGS method to study the function and functional redundancy of the *N. benthamiana* group III E2 genes. We constructed a single chimeric DNA fragment into the pTRV vector by overlap extension PCR and specifically and efficiently knocked down twelve members of group III E2 genes. Assays on the *N. benthamiana* plants where the group III E2 genes were silenced by VIGS revealed the group III E2 genes are essential for plant development, reactive oxygen species (ROS) production, induction of the *NbRbohB* gene, and suppression of immunity-associated programmed cell death (PCD) by AvrPtoB, a type III effector protein of the bacterial pathogen *Pseudomonas syringae* pv. *tomato* (*Pst*) [40]. We revealed that a gene fragment having even two nt mismatched within any 21-bp DNA stretch with the counterpart of another member of the gene family can result in silencing of off-target. This finding is contradicted to the widely-accepted notion that using for VIGS a gene fragment without more than 21 bp of consecutive sequence being identical to other genes in the genome would generate specific gene silencing. We also found that a gene fragment as short as approximately 70 bp in length is sufficient to be used for specific and efficient VIGS. We combined the SGN VIGS tool with manual optimization for the selection of gene fragments for VIGS to specifically knock down a subset of group III E2 members in *N. benthamiana* plants. Characterization of these plants suggested functional redundancy among members of group III E2s in their requirement for immunity-associated ROS production.

## Results

### Utilizing a single chimeric DNA fragment for VIGS to specifically and efficiently knock down twelve members of the highly homologous E2 gene family

**Fig. 1:**
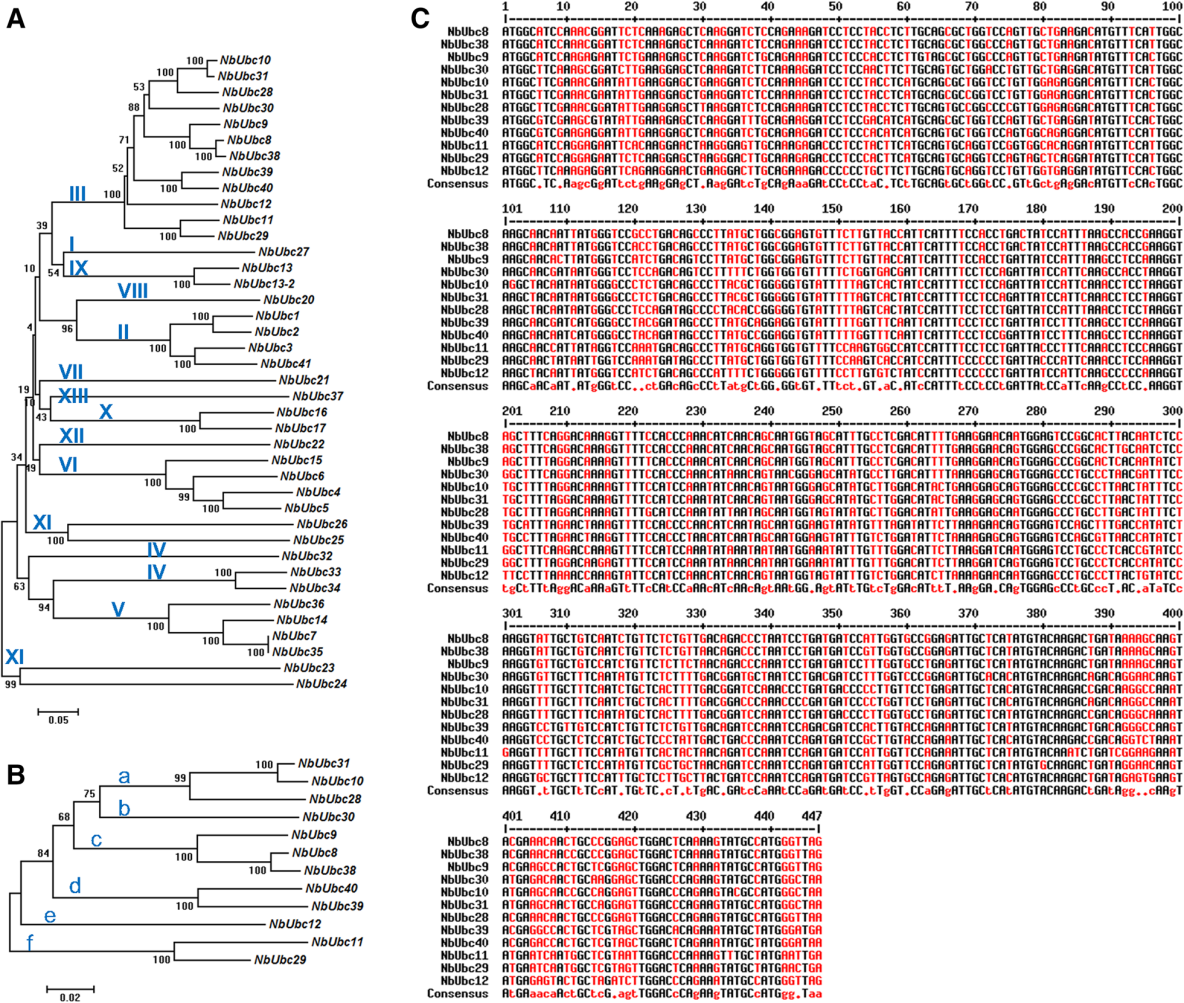
Phylogenetic analysis and sequence alignments of *N. benthamiana* ubiquitin E2 genes. **A** Phylogenetic analysis of 40 *N. benthamiana* ubiquitin E2 genes. The DNA sequences of the open reading frames (ORFs) of 40 *N. benthamiana* E2 genes were used for generating the tree. We used the DNA sequences but not protein sequences to generate the phylogenetic tree to identify most potential off-targets when silencing group III E2 genes using VIGS. The unrooted phylogenetic tree was generated by the neighbor-joining method using the MEGA6 program with 1000 bootstrap trials [61, 68]. The phylogenetic tree was drawn to scale, with branch lengths in the same units as those of the evolutionary distances used to infer the phylogenetic tree. The *Roman numerals* designate the different E2 groups that were based on the phylogenetic analysis of protein sequences as shown in Additional file 1: Fig. S1. **B** Phylogenetic analysis of group III E2 genes. The DNA sequences of the open reading frames (ORFs) of group III E2 genes were used for generating the tree. Same parameters as described in **A** were employed for the phylogenetic analysis. *The letters* (*a*–*f*) designate the six subgroups of group III. **C** Sequence alignments of group III E2 genes. The DNA sequences of the open reading frames (ORFs) of group III E2 genes in the FASTA format were entered into MultAlin and aligned using the default parameters [59]. *Color black* denotes high consensus nucleotide while *red* denotes low and neutral consensus nucleotide

**Fig. 2:**
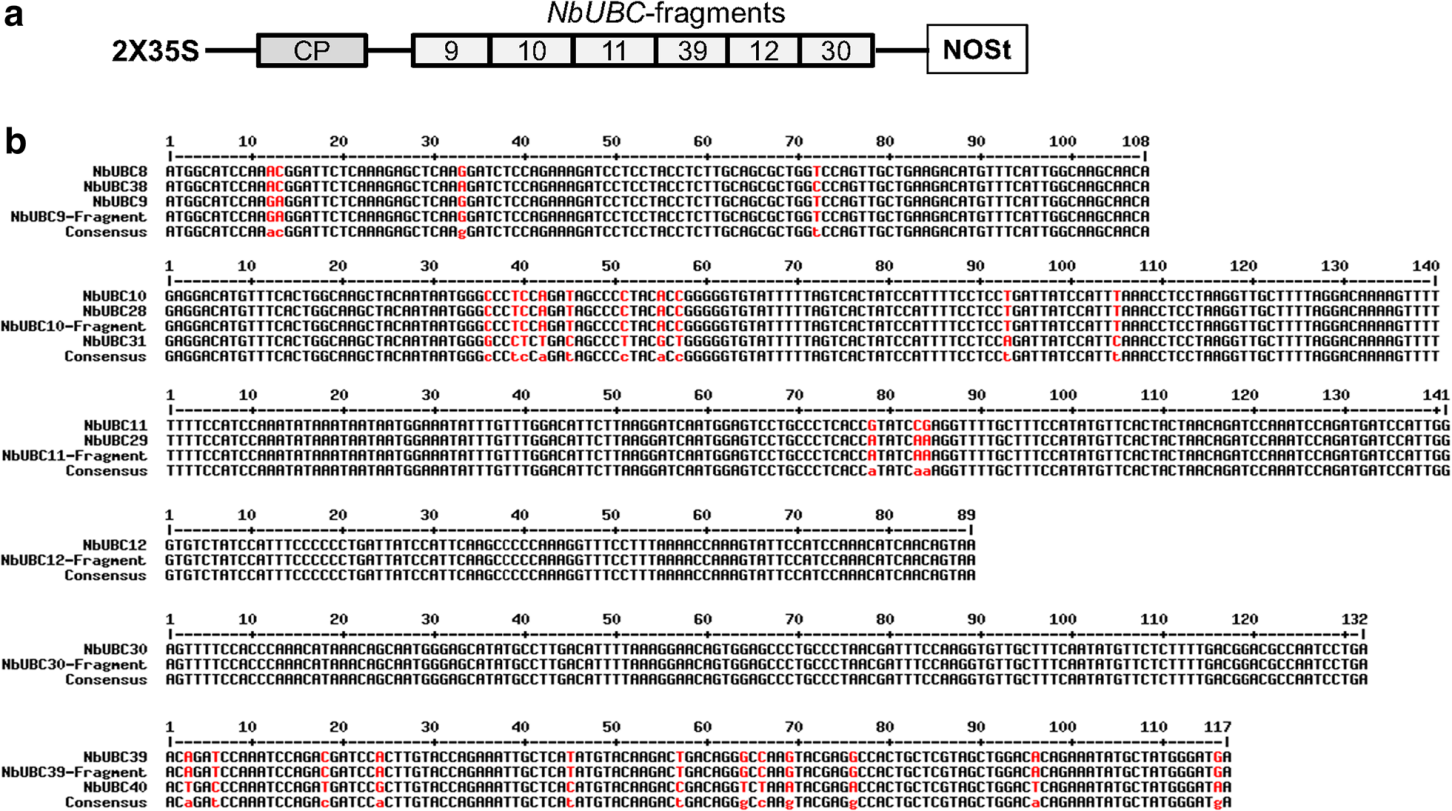
Schematic of the TRV-*III* construct and sequence alignments of the six fragments used for building the TRV-*III* construct with corresponding targeted regions of group III members. **a** Schematic of the TRV-III construct. Six E2 gene fragments were linked by overlap extension PCR and cloned into the TRV2 vector. 2X35S: duplicated 35 S promoters; CP: coat protein of TRV; NOSt: nopaline synthase terminator. The numbers 9, 10, 11, 39, 12 and 30: gene fragment from *NbUBC9*, *10*, *11*, *39*, *12* and *30*, respectively. **b** Sequence alignments of each of the six fragments used for building the TRV-*III* construct with corresponding targeted fragments of group III members. The fragments from *NbUBC12* and *NbUBC30* were designed to silence *NbUBC12* and *NbUBC30*, respectively; the DNA fragment from *NbUBC9* was designed to silence *NbUBC8*, *9* and *38*; the DNA fragment from *NbUBC10* was designed to silence *NbUBC10*, *28* and *31*; the fragment from *NbUBC11* to silence *NbUBC11* and *NbUBC29*; and the fragment from *NbUBC39* to silence *NbUBC39* and *NbUBC40*. Sequences in the FASTA format were entered into MultAlin and aligned using the default parameters [59]. *Color black* denotes high consensus nucleotide while *red* denotes low and neutral consensus nucleotide. The *upper Arabic numerals* designate the positions of nucleotides

We recently identified 40 tomato genes encoding the ubiquitin-conjugating enzymes (E2) and characterized a subset of these genes in plant immunity [39]. Using the tomato E2 genes as the query, we identified forty *N. benthamiana* ubiquitin E2 genes by BLAST [38] (Fig. 1A). Similar to the tomato E2s, the *N. benthamiana* E2 genes can be classified into 13 groups based on phylogenetic analysis of the proteins they encode (Additional file 1: Fig. S1) and group III contains twelve members (Fig. 1A). High sequence identity exists among members of *N. benthamiana* group III E2 genes (Fig. 1B, C). The *N. benthamiana* group III E2 genes can be further divided into six subgroups based on phylogenetic analysis (Fig. 1B, subgroup a–f), of which *NbUBC10*, *28* and *31*; *NbUBC8*, *9* and *38*; *NbUBC11* and *29*; and *NbUBC39* and *40,* respectively each forms a subgroup. The *NbUBC12* and *NbUBC30* show relatively lower identity to other members thus each alone constitutes a subgroup (Fig. 1B). To study the function of group III E2s, VIGS using the TRV-based vector [41, 42] was employed for knocking down the expression of all members in the group in *N. benthamiana*. The insert of the TRV-based construct for silencing the group III E2 genes in *N. benthamiana* (hereafter referred to as TRV-*III*) was prepared by overlap extension PCR to link six individual fragments that are derived from *NbUBC9*, *NbUBC10*, *NbUBC11, NbUBC12*, *NbUBC30* and *NbUBC39*, respectively (Fig. 2a). Due to high percentage of identity in their DNA sequences, a DNA fragment derived from *NbUBC9* can theoretically be used for silencing the *NbUBC8*, *NbUBC9* and *NbUBC38* genes that are from the same clade of the phylogenetic tree (Fig. 1B) and was thus designed for building the TRV-*III* construct. Similarly, a DNA fragment from *NbUBC10* was designed for silencing genes *NbUBC10*, *NbUBC28* and *NbUBC31*, a fragment from *NbUBC11* was designed for silencing of *NbUBC11* and *NbUBC29*, and a fragment from *NbUBC39* for silencing of *NbUBC39* and *NbUBC40* (Fig. 2b).

**Fig. 3:**
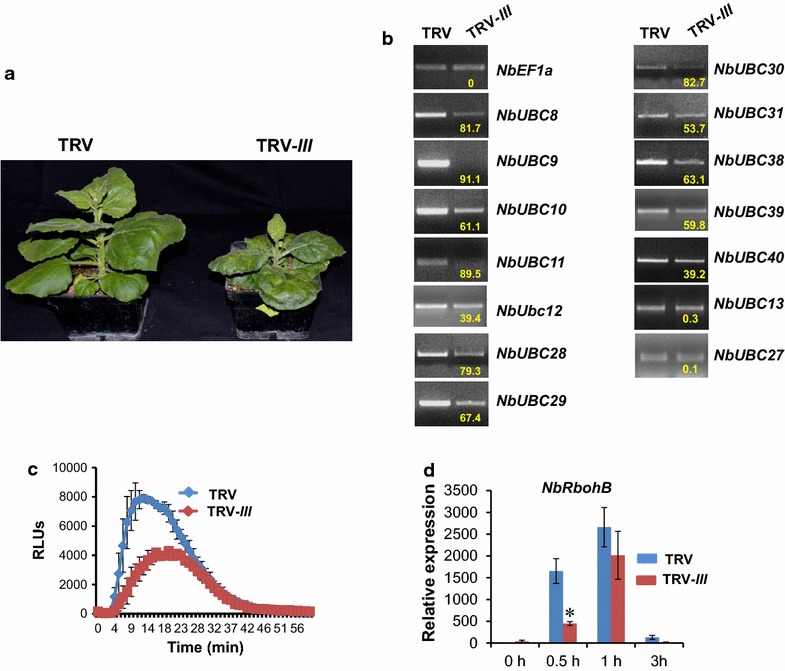
The group III ubiquitin E2 s are required for ROS production and the induction of *NbRbohB*. **a** Phenotype of the *N. benthamiana* plants in which the group III E2 genes were silenced. The non-silenced TRV empty vector (TRV)-infected plant was used as control. Photographs were taken 4 weeks after the approximately 3-week old seedlings were infiltrated with TRV or TRV-*III* VIGS constructs. **b** The group III ubiquitin E2 genes were efficiently and specifically silenced in *N. benthamiana* by VIGS. The transcript level of group III E2 genes and closely-related E2 genes *NbUBC13* and *NbUBC27* (outside the group III) in non-silenced TRV control (TRV) and group III E2 genes-silenced (TRV-*III*) *N. benthamiana* plants was examined by semi-quantitative PCR. *NbEF1α* was used as an internal reference for determining the amount of cDNA template to be used. *Numbers* under the gel bands denote the percentage of reduction (%) in expression of the corresponding gene in TRV-*III*-infected plants compared to that in control plants. Experiments were repeated three times with similar results. **c** Silencing the group III E2 genes resulted in reduced ROS production induced by flg22 in a chemiluminescence assay. Leaf discs of the group III E2 genes-silenced (TRV-*III*) and non-silenced TRV control (TRV) *N. benthamiana* plants were incubated with 1.0 μM flg22 to induce ROS production. The experiment was repeated three times with similar results. *Error bars* indicate standard deviation. **d** Knocking down the group III ubiquitin E2 genes down-regulates the induction of gene *NbRbohB* by flg22. The expression of *NbRbohB* in 2.0 μM flg22-treated leaves of group III E2 genes-silenced and non-silenced TRV control *N. benthamiana* plants was analyzed by real time PCR at the indicated time points after flg22 treatment. The X-axis marks different time points (hour) after flg22 treatment. The experiment was performed with three technical repeats in each of the three biological replicates. *Error bars* indicate standard deviation. *Asterisks* mark significant reduction of the expression of *NbRbohB* in group III E2 genes-silenced plants compared with that in non-silenced control plants based on one-way ANOVA (P < 0.01)

VIGS on *N. benthamiana* plants was performed as previously described [43] using the *Agrobacterium tumefaciens* strain GV2260 containing the plasmid of empty TRV vector and TRV-*III*, respectively. The *N. benthamiana* plants infected with TRV-*III* displayed slower growth and thus were smaller than the control plants, which indicates the group III E2 enzymes are required for plant development. They also showed slightly crinkled leaves (Fig. 3a). To determine the effectiveness and specificity of knocking down group III E2 genes by VIGS in *N. benthamiana*, the expression of 12 group III E2 genes was determined by semi-quantitative PCR. The results showed that *N. benthamiana* plants infected with TRV-*III* displayed greatly reduced expression of group III E2 genes compared with TRV empty vector-infected plants (Fig. 3b). By contrast, the expression level of genes encoding the highly homologous, non-group III E2 enzymes UBC13 and 27 (Fig. 1A) was not altered in TRV-*III* infected plants as compared with the TRV-infected plants, which suggests the silencing of group III E2 genes by TRV-*III* is specific.

### Group III E2s were essential for ROS production and the induction of *NbRbohB*

Pathogen-associated molecular pattern (PAMP) can induce rapid and transient ROS production in plants in an oxidative burst shortly after treatment, which is an early plant immune response to pathogen infection [44]. To test the effect of silencing group III E2 genes on plant immunity, we examined the ROS production upon treatment of leaves of TRV-*III* infected and TRV-infected *N. benthamiana* plants, respectively by flg22, a 22-amino-acid immunogenic fragment of flagellin that is the major PAMP of many pathogenic bacteria. Compared with TRV-infected *N. benthamiana* plants, the ROS production triggered by flg22 treatment was significantly reduced on TRV-*III*-infected *N. benthamiana* plants (Fig. 3c), suggesting group III E2 enzymes play a positive role in the induction of PTI-associated ROS production. In Arabidopsis, the respiratory burst oxidase homologues (*Rboh*) genes encoding the NADPH oxidase, *AtRbohD* and *AtRbohF* were found to be required for the generation of plant immunity-associated ROS [45]. The *N. benthamiana* gene *NbRbohB*, an ortholog of *AtRbohD*, has been shown to be responsible for the ROS production induced by flg22 [46]. Silencing of *NbRbohB* abrogates the flg22- and chitin-induced ROS burst in *N. benthamiana* [47]. To further confirm the role of group III E2 genes in flg22-triggered ROS production, we challenged the leaves of TRV-*III*- and TRV-infected *N. benthamiana* plants, respectively with flg22 and monitored the expression of the *NbRbohB* gene at 0 (before inoculation), 0.5, 1 and 3 h post treatment. In the TRV control plants, the expression of *NbRbohB* was significantly induced 0.5 and 1 h after flg22 treatment (HAT) (Fig. 3d). Compared to control, the induction of *NbRbohB* at 0.5 HAT was significantly reduced on plants in which the group III E2 genes were knocked down.

### Knocking down of group III E2 genes diminished the inhibition of immunity-associated PCD by AvrPtoB in *N. benthamiana*

**Fig. 4:**
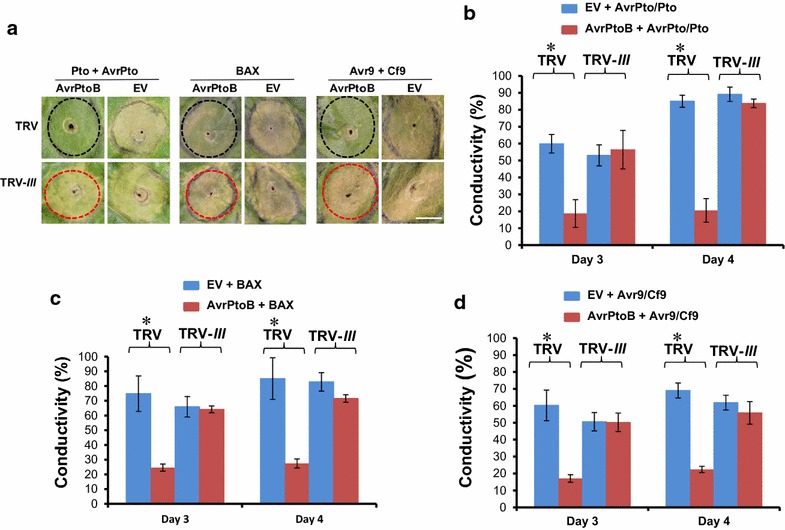
The suppression of PCD by AvrPtoB in non-silenced (TRV) and group III E2 genes-silenced (TRV-*III*) *N. benthamiana* plants. **a** Silencing the group III E2 genes diminishes the suppression of AvrPto + Pto, BAX, and Avr9 + Cf9-elicited PCD by AvrPtoB in *N. benthamiana*. Transient co-expression of AvrPtoB and PCD elicitors in fully expanded *N. benthamiana* leaves was performed by *Agro*-infiltration. Transient co-expression of the empty pBTEX vector (EV) and the PCD elicitors was used as control. Reduced suppression of PCD by AvrPtoB on group III E2 genes-silenced plants compared to TRV control plants are marked by *red* and *black dash*-*line circles*, respectively. Photographs were taken on day four after infiltration. The experiment was repeated at least two times with similar results. **b–d** Examination of the suppression of PCD by AvrPtoB in non-silenced (TRV) and group III E2 genes-silenced (TRV-*III*) *N. benthamiana* plants using electrolyte leakage assays. Transient co-expression of AvrPtoB and the PCD elicitor (AvrPto + Pto, BAX, or Avr9 + Cf9) in fully expanded *N. benthamiana* leaves was performed as described in **a**. The percentage of conductivity measured on the third and fourth day compared to the total conductivity was shown. *Asterisks* mark significant difference between the two treatments based on one-way ANOVA (P < 0.01). The experiment was repeated at least three times with similar results

AvrPtoB has been shown to suppress PCD mediated by immunity-associated cell death elicitors AvrPto + Pto, BAX and Avr9 + Cf9 [48]. Our recent study of the group III E2 enzymes indicated that they are required for the suppression of Fen-mediated PCD by AvrPtoB and AvrPtoB-directed ubiquitination of Fen [39]. However, whether group III E2 genes are also essential for the suppression of other immunity-associated PCD by AvrPtoB is unknown. To address this question, the induction of PCD was examined by co-expression of AvrPtoB and the PCD elicitors AvrPto + Pto, BAX, and Avr9 + Cf9 on TRV-*III*- and TRV- infected *N. benthamiana* plants, respectively. Co-expression of empty vector and the PCD elicitors was employed as control. The results indicated AvrPtoB suppresses PCD trigged by these elicitors on TRV control plants, which is consistent with the previous finding [48]. However, knocking down of group III E2 genes diminished AvrPtoB-mediated suppression of PCD elicited by AvrPto + Pto, BAX, and Avr9 + Cf9 (Fig. 4a). The visual observation of reduction in AvrPtoB-mediated suppression of PCD by these elicitors on TRV-*III*-treated plants were substantiated by electrolyte leakage assays, as shown in Fig. 4b, c and d.

### Four members of the *N. benthamiana* group III E2 were specifically and efficiently knocked down by improved selection of gene fragments for VIGS

**Fig. 5:**
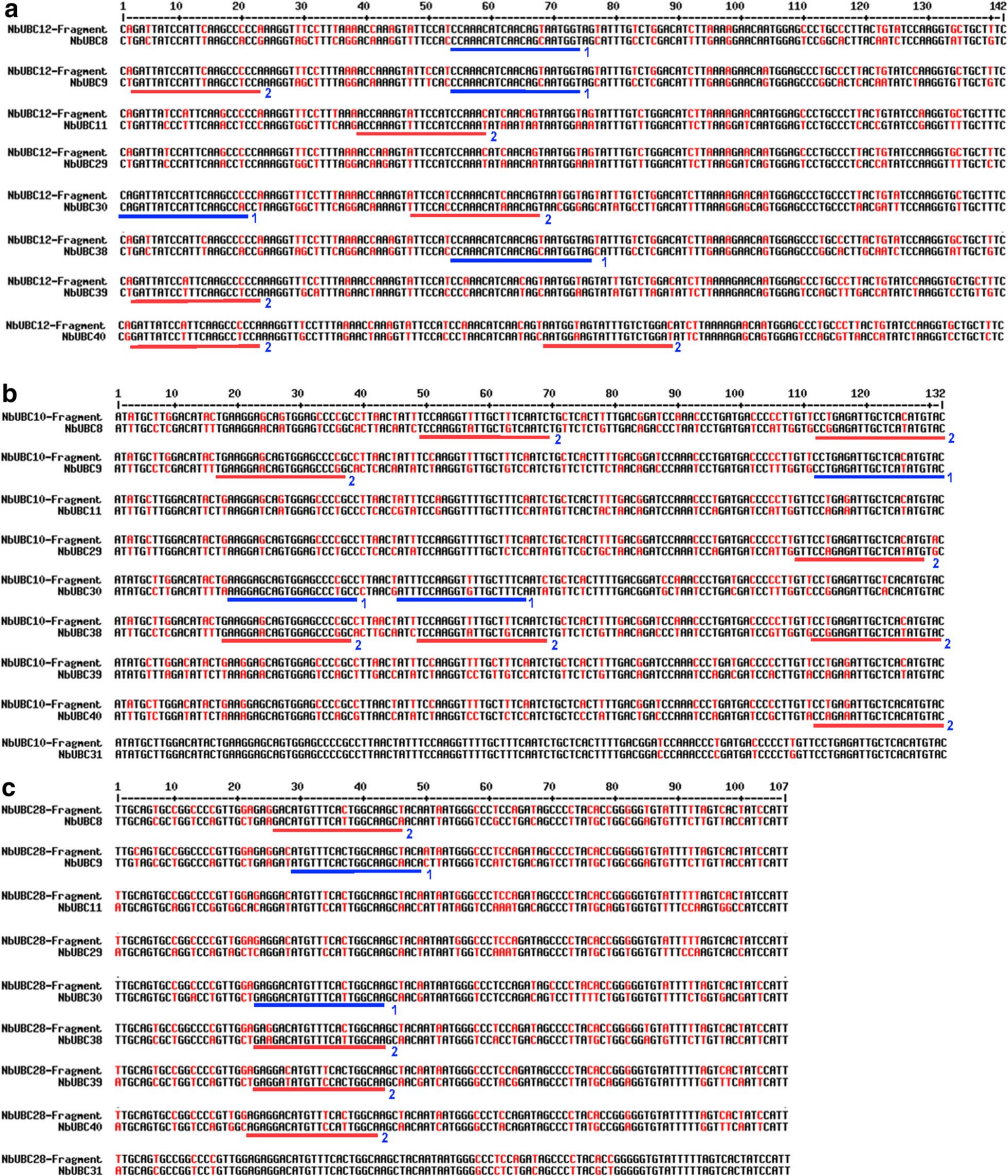
Sequence alignments of the three gene fragments used for building the VIGS construct TRV-*UBC12/10/28/31* and the corresponding region of other group III E2 members. The alignments of gene fragments from *NbUBC12* (**a**), *NbUBC10* (**b**), and *NbUBC28* (**c**), respectively with the corresponding region from *NbUBC8*, *9*, *11*, *29, 30*, *38*, *39* and *40* were shown. The DNA fragments from *NbUBC10* and *NbUBC28* were designed to silence *NbUBC10*, *28* and *31*. *Color black* denotes high consensus while *red* denotes low and neutral consensus. The *upper Arabic numerals* designate the positions of the nucleotides. The sequences *underlined* in *blue* were 21-bp DNA stretches within which one nt is mismatched. The sequences underlined in *red* were 21-bp DNA stretches within which two nt are mismatched. *Numbers* at the *right* of the *underlines* denote the counts of mismatched nucleotides in the 21-bp DNA stretches. The non-underlined regions of each alignment have more than 2 mismatched nt within any 21-bp DNA stretches

**Fig. 6:**
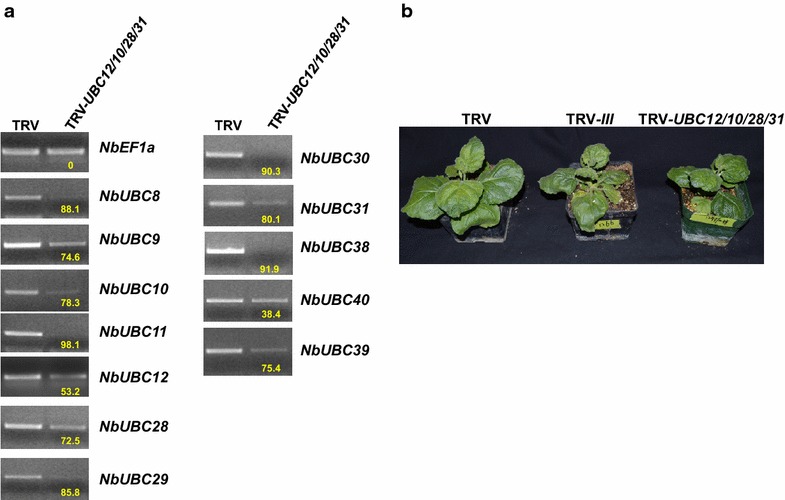
The silencing of off-targets in TRV-*UBC12/10/28/31*-infected *N. benthamiana* plants. **a** The expression level of group III E2 genes in TRV-*UBC12/10/28/31*-infected *N. benthamiana* plants. The transcript level of group III E2 genes in non-silenced TRV control (TRV) and TRV-*UBC12/10/28/31*-infected *N. benthamiana* plants was examined by semi-quantitative PCR. *NbEF1α* was used as an internal reference for determining the amount of cDNA template to be used. Numbers under the gel bands denote the percentage of reduction (%) in the expression of corresponding gene in TRV-*III*-infected plants compared to that in non-silenced TRV control plants. **b** Phenotype of the *N. benthamiana* plants in TRV-*UBC12/10/28/31*-infected *N. benthamiana* plants. The non-silenced TRV empty-vector (TRV) and group III E2-silenced plants were used as control. Photographs were taken 4 weeks after the approximately 3-week old seedlings were infiltrated with the TRV-*UBC12/10/28/31* construct, the TRV-*III* construct, and the TRV empty vector, respectively. The experiment was repeated two times with similar result

The group III members of *N. benthamiana* E2 gene family are highly homologous (Fig. 1C), which promoted us to find out whether functional redundancy exists among the twelve genes in their requirement for plant immunity-associated ROS production. Analysis of the transcript level in tomato plants treated with a *Pst* DC3000 strain delivering AvrPtoB indicated that among the twelve tomato group III E2 genes, only *UBC10*, *12*, *28* and *31* were induced after the *Pst* treatment [39]. We thus attempted to examine the role of the corresponding *N. benthamiana* genes *UBC12*, *10*, *28* and *31* in plant immunity by only silencing these four genes using VIGS. The sequence specificity of the RNAi process is thought to be determined by the 21- to 24-nt siRNAs generated by the RNA-induced silencing complex (RISC) [49]. To avoid silencing of off-targets because of extremely high homology among members of the group III E2 genes, we first utilized the SGN VIGS Tool software to identify regions of the *NbUBC12*, *10*, *28* and *31* genes, respectively that have highest mismatches to the DNA sequence of other group III E2 members. The regions identified by the SGN VIGS Tool software were further scrutinized manually to ensure at least one nt is mismatched to the corresponding sequence of other members within any consecutive 21-bp DNA stretch. The unqualified sequences (i.e. do not contains at least one mismatched nucleotide to other genes within any 21-bp sequence) of the fragments identified by the SGN VIGS Tool were excluded. The final fragment for building the TRV-*NbUBC12/10/28/31* construct was cloned by overlap extension PCR to link three individual fragments derived from *NbUBC12*, *NbUBC10* and *NbUBC28*, respectively (Fig. 5). Alignments of the fragments derived from *NbUBC12*, *NbUBC10* and *NbUBC28*, respectively to the corresponding region of other members of the group III E2s suggested that the DNA fragment from *NbUBC12* would theoretically target *NbUBC12* only in VIGS while the fragments from *NbUBC10* and *NbUBC28* would target *NbUBC10*, *28* and *31* for silencing. VIGS using this construct was then performed on *N. benthamiana* plants via *Agrobacterium*-mediated infiltration as described [43]. Plants infected with the empty TRV vector were used as control. The expression of group III E2 genes examined by semi-quantitative PCR, however, indicated all members of the group III E2 genes were knocked down, which suggests silencing of off-targets and thus low specificity occurred during VIGS using the TRV-*NbUBC12/10/28/31* construct (Fig. 6a). In consistence with this observation, the *N. benthamiana* plants infected with TRV-*NbUBC12/10/28/31* displayed similar phenotype in growth and development to the TRV-*III* infected plants (Fig. 6b).

**Fig. 7:**
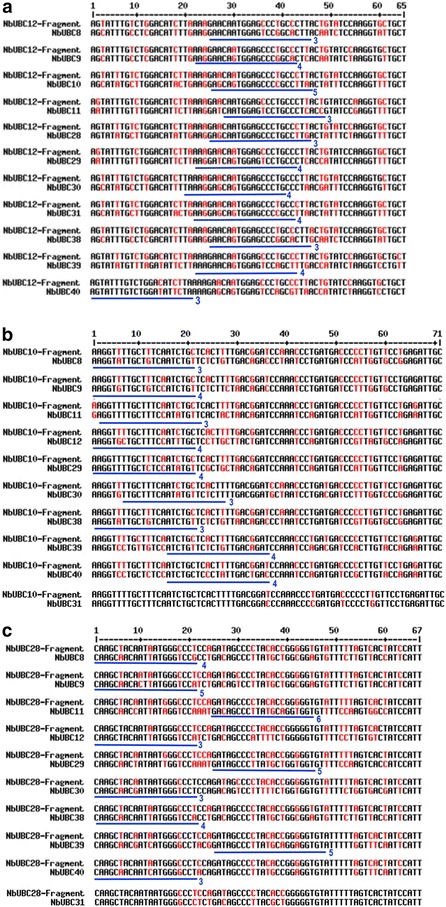
Sequence alignments of three re-selected gene fragments used for silencing *NbUBC12*, *10*, *28* and *31* and corresponding fragments of other group III E2 genes. The DNA fragments selected by SGN tool for VIGS were further optimized to ensure at least 3 nt mismatches within any 21-nt DNA stretch. The DNA fragment from *NbUBC12* was designed to silence *NbUBC12*; the DNA fragments from *NbUBC10* and *NbUBC28* were designed to silence *NbUBC10*, *28* and *31*. The alignments of optimized DNA fragments from *NbUBC12* (**a**), *NbUBC10* (**b**), and *NbUBC28* (**c**), respectively with the corresponding region from *NbUBC8*, *9*, *11*, *29, 30*, *38*, *39* and *40* were shown. *Color black* denotes high consensus nucleotide while *red* denotes low and neutral consensus nucleotide. The sequences *underlined* in *blue* were the 21-bp DNA stretches within which at least 3 nt are mismatched. *Numbers* at the *right* of the *underlines* denote the counts of mismatched nucleotides in 21-bp DNA stretches

**Fig. 8:**
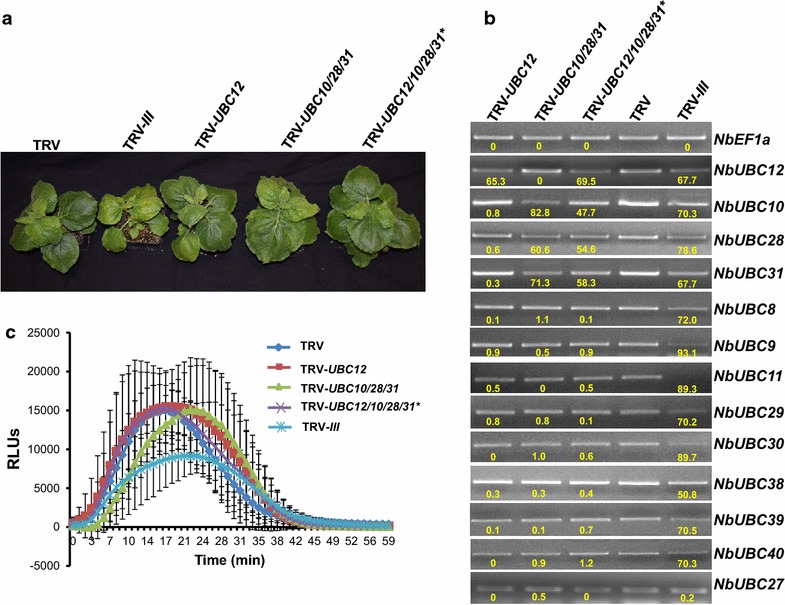
Specific and efficient knocking-down of four members of group III E2 genes did not significantly affect flg22-induced ROS production and plant development. **a** Phenotypes of the *N. benthamiana* plants in which *NbUBC12* alone, three E2 genes *NbUBC10*, *28* and *31*, or four E2 genes *NbUBC10, 12*, *28* and *31* were specifically silenced. The optimized VIGS construct TRV-UBC12/10/28/31* was used to specifically silence *NbUBC10, 12*, *28* and *31.* The non-silenced TRV-infected plant and group III-silenced plant were included as control. Photographs were taken 4 weeks after the approximately 3-week-old seedlings were infiltrated with TRV vector-based VIGS constructs. **b** The *NbUBC10*, *12*, *28* and *31* genes were specifically and efficiently silenced in *N. benthamiana* by VIGS. The transcript level of group III E2 genes and a closely-related E2 gene, *NbUBC27* (outside the group III) in various VIGS-treated *N. benthamiana* plants was examined as described. *NbEF1α* was used as an internal reference for determining the amount of cDNA template to be used. *Numbers* under the *gel bands* denote the percentage of reduction (%) in the expression of corresponding gene in plants treated with TRV-*UBC12*, TRV-*UBC10/28/31*, and TRV-UBC12/10/28/31*, respectively compared to that in non-silenced TRV control plants. The experiments were repeated three times with similar results. **c** Knocking down *NbUBC10*, *12*, *28* and *31* genes did not affect significantly flg22-induced ROS production. Leaf disks of the VIGS-treated *N. benthamiana* plants were incubated with 1.0 μM flg22 to induce ROS production. *Error bars* indicate standard deviation. Experiments were repeated three times with similar results

To figure out the cause of low specificity, we reviewed the sequence alignments as shown in Fig. 5 and counted the numbers of 21-bp DNA stretches containing one or two nt mismatches to the corresponding region of other eight group III E2 genes. As summarized in Table 1, such mismatches exist between other eight members, *NbUBC8*, *9*, *11*, *29, 30*, *38*, *39* and *40* in group III and at least one of the three fragments that were derived from *NbUBC12*, *NbUBC10* and *NbUBC28*, respectively for VIGS. For *NbUBC11*, *29, 39* and *40*, only 21-bp DNA stretches with two nt mismatches were identified. Yet these genes were silenced by the TRV-*NbUBC12/10/28/31* construct, which suggests that even mismatches at two nt within a consecutive 21-bp sequence can still result in silencing of off-targets in VIGS. Bearing this in mind, we improved our method for building the construct for VIGS. Similarly, we first utilized the SGN VIGS Tool for the preliminary selection of VIGS fragments. The SGN VIGS Tool can only identify DNA fragments that are equal to or are more than 100 bp and it failed to pinpoint a region of the gene-of-interest (e.g. *NbUBC12*) that has more than 2 nt mismatched within any consecutive 21-bp sequence to other members of the group III E2 genes. Therefore, we next manually excluded some sequences from the preliminary fragments identified by the SGN VIGS Tool and minimized the VIGS fragment to ~70 bp to ensure mismatches of at least 3 nt exist within any consecutive 21-bp of the shortened fragment to the sequences of other eight group III E2 genes. In attempting to uncover parameters that govern the functional miRNA:mRNA interactions, a previous study has generated a set of rules for the evaluation of putative targets of a miRNA [50]. In general, paring in the 5′ part of the miRNA to the target mRNA is most important. Only one mismatch would be allowed in the regions corresponding to the nucleotides 2–12, which includes the presumptive cleavage site between positions 10 and 11. For pairing in the 3′ end of the miRNA, a mismatch loop could be tolerated up to maximal two nucleotides, and a perfect match in this part would compensate the presence of up to two mismatches in the 5′ end. Considering that TRV-*NbUBC12/10/28/31*-generated siRNAs with two nt mismatched to the off-target mRNAs might have downregulated the off-target gene using a mechanism similar to that of miRNA, we also avoided selecting gene regions that may result in a functional miRNA that targets other group III E2 genes. The alignments of new individual DNA fragments that we used for building the new TRV-based construct (TRV-NbUBC12/10/28/31*) with the corresponding region of other eight members of the group III E2s are shown in Fig. 7. The sequences underlined in blue were the DNA stretches within which at least 3 nt mismatch exists in any consecutive 21-bp DNA sequence (Fig. 7). The VIGS constructs TRV-*NbUBC12*, TRV-*NbUBC10/28/31* and TRV-NbUBC12/10/28/31* prepared by this improved method were used to silence *NbUBC12* alone, the three E2 genes *NbUBC10*, *28* and *31*, and the four E2 genes *NbUBC12*, *10*, *28* and *31*, respectively. The *N. benthamiana* plants infected with TRV-*NbUBC12*, TRV-*NbUBC10/28/31* and TRV-NbUBC12/10/28/31* displayed similar phenotype to TRV-infected control plants (Fig. 8a). To determine the effectiveness and specificity of knocking down the four genes by VIGS in *N. benthamiana*, semi-quantitative PCR was performed to detect the level of expression for all twelve group III genes and *NbUBC27*. The results indicated *UBC12* alone; the three genes *UBC10*, *28* and *31*; and the four genes *UBC10*, *12*, *28*, and *31*, respectively were specifically silenced in *N. benthamiana* plants (Fig. 8b).

**Table 1:** Numbers of sites at which one or two nt are mismatched within a 21-bp sequence region in the alignments of individual gene fragment to be used for building the TRV-*UBC12/10/28/31* VIGS construct and corresponding region of other eight group III members

E2	*NbUBC12* fragment		*NbUBC10* fragment		*NbUBC28* fragment	
	1/21	2/21	1/21	2/21	1/21	2/21
*NbUBC8*	1	0	0	2	0	1
*NbUBC9*	1	1	1	1	1	0
*NbUBC10*	–	–	–	–	–	–
*NbUBC11*	0	1	0	0	0	0
*NbUBC12*	–	–	–	–	–	–
*NbUBC28*	–	–	–	–	–	–
*NbUBC29*	0	0	0	1	0	0
*NbUBC30*	1	1	2	1	1	0
*NbUBC31*	–	–	–	–	–	–
*NbUBC38*	1	0	0	3	0	1
*NbUBC39*	0	1	0	0	0	1
*NbUBC40*	0	2	0	1	0	1

### Knocking down a subset of group III E2 members did not result in significant changes in plant ROS production

The ROS production after treatment with flg22 on leaves of *N. benthamiana* plants infected with TRV-*NbUBC12*, TRV-*NbUBC10/28/31* and TRV-NbUBC12/10/28/31* was monitored. Compared with TRV-infected *N. benthamiana* plants, the ROS production triggered by flg22 treatment was similar on TRV-*NbUBC12*, TRV-*NbUBC10/28/31* and TRV-NbUBC12/10/28/31* N. benthamiana plants (Fig. 8c), suggesting functional redundancy for ROS production existed among members of group III.

## Discussion

VIGS has been widely used in the past decade as a fast and efficient functional genomics tool for knocking down gene expression in many plant species [51, 52]. Advantages of VIGS over other functional genomics tools include the easiness and quickness of the method, no need of stable plant transformation, knowledge of partial sequence of a gene is sufficient to be used with the method, and it can be used in both forward and reverse genetics [52]. Because VIGS is a sequence homology-dependent process, another significant advantage of VIGS is that it can be used to knock down simultaneously the expression of multiple members of a gene family by targeting highly conserved sequence shared by the members. Moreover, because knocking down but not knocking out of a gene usually occurs during VIGS, it can thus be used to study the function of genes of which knocking out would cause lethality of the plant. In addition to silencing gene systematically on the whole plant, VIGS has also been applied to knock down gene expression in specific plant organs [25, 27, 28]. Despite these benefits and its wide application to the study of gene function, however, there are inherent potential limitations of VIGS, such as silencing of off-targets [52]. The unintended silencing of off-target genes can lead to false conclusions in VIGS experiments. It is thus important to choose a gene fragment that would target the gene-of-interest specifically.

Theoretically, silencing of off-target genes in VIGS can be avoided by selecting a gene fragment that does not produce 21- to 24-nt siRNAs being identical in sequence to other gene(s) in the genome [53]. Nevertheless, a previous study on *N. benthamiana* and other Solanaceae species suggested that 21-nt DNA stretch of 100% identity between the gene-of-interest and off-target gene sequences is not absolutely required for silencing of off-target genes [54]. The study indicated even a continuous 11-nt identical DNA stretch between the gene fragment used for VIGS and the homologous gene in another species can lead to ~15% reduction in the expression of the homologues. In the first tries of the present study, we attempted to knock down by VIGS four members of group III E2s, *NbUBC12*, *10*, *28* and *31*, by ensuring at least one nt mismatch to other group III members within any 21-bp DNA stretch in the gene fragments used for building the TRV-*NbUBC12/10/28/31* construct. However, our result suggested that even 2-nt mismatches within a 21-bp DNA stretch can result in silencing of off-targets. In plants, miRNAs are another kind of small non-coding RNA molecule in the length of 21–24 nt that functions in gene silencing [55]. Most plant miRNAs interact with their mRNA targets with nearly perfect complementarity except bulges or mismatches present in the central region of the miRNA-mRNA duplex [56]. A microarray analysis of artificial miRNAs revealed a set of rules for evaluation of a putative miRNA target, in which 2 or 3 nt mismatch are allowed to be present at some sites [50]. Silencing of off-target genes observed in the present and the previous studies [54] thus might be due to that siRNAs with 2 nt mismatched to the off-target mRNAs downregulated the off-target genes using a mechanism similar to that of miRNA. Indeed, mismatched siRNAs have been proved to be able to downregulate mRNAs, though mismatched siRNAs targeting the 3′ UTRs yield a greater reduction in mRNA levels than those targeting the coding region [57]. Based on the result of our first tries and findings from other studies, we improved our method to knock down the four members of group III E2s. Through combining the use of SGN VIGS tool, manual selection of the gene fragments for VIGS, and following the set of rules for evaluation of a functional miRNA [50], we used gene fragments with at least 3 nt mismatch within any 21-bp DNA stretch to other members of the group to specifically and efficiently knock down the expression of a subset of members of the group III E2 gene family. Our results using the new VIGS construct suggested that using such gene fragments for VIGS prevents the silencing of off-targets. Generally, the optimum length of gene fragment for efficient VIGS with minimal off-target effects was thought to be about 200–400 nt [53]. However, our result indicated that VIGS using TRV vectors carrying gene fragments in the length of about 70 bp resulted in efficient gene silencing. Therefore, shorter fragments under 100 bp can be used for efficient gene silencing in VIGS.

VIGS can be used to silence multiple members of a gene family by targeting highly conserved sequence regions of the members [52]. In line with this, we used VIGS as an effective tool for functional study of members of a gene family in this study. By constructing a single chimeric gene fragment for VIGS using overlap extension PCR to link multiple gene fragments together, we successfully targeted twelve members and four members of group III E2 genes, respectively. Our results indicated that group III E2s are required for plant growth and development, PTI-associated reactive oxygen species (ROS) production, and induction of the *NbRbohB* gene that is essential for ROS production. We also found that these E2 genes are essential to the suppression of multiple immunity-associated programmed cell death (PCD) by AvrPtoB, which suggests an important role for group III E2s in AvrPtoB-mediated suppression of plant immunity during the interaction of tomato with *Pst*. The functional redundancy among group III E2 members as shown on the *N. benthamiana* plants in which the expression of *NbUBC12*, *10*, *28* and *31* of group III E2 genes was downregulated by VIGS highlights the necessity of using VIGS to silence the whole group III E2 genes in order to elucidate the role of these genes in plant development and immunity. Such results, in turn, indicate the feasibility of the improved VIGS method in the study of highly homologous members in a gene family.

## Conclusions

In this study, we use an improved virus-induced gene silencing approach to efficiently silence multiple highly homologous members of a gene family meanwhile prevent the silencing of off-targets, a highly concerned potential problem inherent with VIGS. The improved approach involves enhanced selection and evaluation of gene fragments used for VIGS. Additionally, we found that a gene fragment as short as about 70 bp can be used for VIGS to silence gene-of-interest efficiently. Using this method, we specifically and efficiently knocked down twelve and four members of a highly homologous gene family, respectively. Assays using *N. benthamiana* plants where the target genes were knocked down by this improved VIGS approach revealed that group III E2s are essential for plant development, reactive oxygen species (ROS) accumulation, induction of the gene *NbRbohB*, and suppression of multiple immunity-associated PCD by AvrPtoB. Additionally, through specific knocking-down of four members of group III E2 genes we identified functional redundancy among group III members in their requirement for plant development and plant immunity-associated ROS production.

## Methods

### Growth of bacteria and plant materials

*Agrobacterium tumefaciens* GV2260 strains were grown at 28 °C on Luria–Bertani and King’s B medium with appropriate antibiotics. *N. benthamiana* seeds were germinated and grown on autoclaved soil in a growth chamber with 16 h light (~300 μmol/m^2^/s at the leaf surface of the plants), 24/23 °C day/night temperature, and 50% relative humidity.

### DNA manipulations and plasmid constructions

All DNA manipulations were performed using standard techniques [58]. The chimeric DNA fragment of the TRV-*III* construct used for silencing the group III E2 genes in *N. benthamiana* was cloned by overlap extension PCR by which six individual gene fragments from the genes *NbUBC9*, *11*, *12*, *10*, *30* and *39*, respectively were linked together. Scrutiny of sequences was conducted during the selection of DNA fragments for building the TRV-*III* construct to avoid silencing of off-target E2 genes from other groups. TRV-*III* construct for VIGS was prepared by Gateway cloning using the pENTR/SD/D-TOPO entry vector and the pTRV2 vector [42] according to protocols provided by the manufacturer (Life Technologies). For testing functional redundancy of group III members, fragments used for building the VIGS constructs to silence the E2 genes *NbUBC12, 10*, *28* and *31* were first cloned into pENTR/SD/D-TOPO entry vector, and then cloned into pTRV2 by LR reaction using protocols provided by the manufacturer (Life Technologies). Primers used for recombinant DNA cloning are listed in the Additional file 2: Table 1.

### Sequence alignments and phylogenetic analysis

For sequence alignments of group III E2 genes and DNA fragments to be used for VIGS, sequences in the FASTA format were entered into MultAlin [59], a multiple sequence alignment program and aligned using the default parameters. For phylogenetic analysis, sequences of interest in the FASTA format were loaded into the ClustalX 2.1 program and aligned using the ClustalX algorithm [60]. The phylogenetic analysis was then performed by the MEGA6 program using the aligned sequences [61]. To build an unrooted phylogenetic tree using MEGA6, the evolutionary history was inferred using the neighbor-joining method with 1000 bootstrap trials. The evolutionary distances were computed using the p-distance method in which the evolutionary distance unit represents the number of amino acid (or nucleotide) substitutions per site [62]. Branches corresponding to partitions reproduced <50% bootstrap replicates were collapsed in the tree. The sequence IDs of the *N. benthamiana* ubiquitin E2 genes in the sol genomics network (http://solgenomics.net/) database are as shown previously [39].

### Effectiveness and specificity of VIGS of group III E2 genes

To determine the effectiveness of knocking down group III E2 genes by VIGS in *N. benthamiana*, the expression of these genes was determined by semi-quantitative PCR at low PCR cycles. In brief, six leaf discs comprising a total area of ~3.8-cm^2^ were collected from three different leaves of three VIGS-treated plants (one leaf of each plant). Total RNA was then isolated using RNeasy Plant Mini Kit (QIAGEN) and treated with DNase (QIAGEN) by following the protocol provided by the manufacturer. The first-strand cDNA was synthesized using the Superscript III reverse transcriptase (Life Technologies) by following the instructions provided by the manufacturer. Examination of the transcript level of the *N. benthamiana* E2 genes *NbUBC8*, *NbUBC9*, *NbUBC10*, *NbUBC11*, *NbUBC12*, *NbUBC28*, *NbUBC29*, *NbUBC30*, *NbUBC31*, *NbUBC38*, *NbUBC39*, *NbUBC40* and *NbEF1a* by PCR was conducted using the first-strand cDNA as template and the primers listed in the Additional file 2: Table 1. The PCR products were resolved by 1.2% agarose gel. The separated PCR products were photographed using the Gel Doc SR system (Bio-Rad). *NbEF1a* was used as an internal reference to determine the amount of the first-strand cDNA template to be used. The percentage of reduction in expression of corresponding gene in group III E2 genes-silenced plants compared to non-silenced TRV control plants was calculated based on the pixel density of bands using the Gel Analysis option of the ImageJ software [63]. The primers for semi-quantitative PCR were shown in Additional file 2: Table 1.

### Measurement of ROS production

The ROS production assay was used to quantify the induction of ROS as described previously [64] with minor modifications. In brief, leaf discs of 4 mm in diameter were punched out from *N. benthamiana* plants with a cork borer and floated adaxial side up in 200 µL deionized pure H_2_O at room temperature overnight in wells of a 96-well Nunc white plate (one leaf disc per well) (Thermo Scientific). On the second day, the deionized pure H_2_O was replaced with 100 µL of ROS testing buffer containing 1 µM of flg22 peptide, 34 mg/mL of luminol (Sigma), and 20 μg/mL of horseradish peroxidase (Sigma). Luminescence was measured using a Bioteck Synergy II plate reader. Twelve leaf discs from three group III E2 genes-silenced and three non-silenced control *N. benthamiana* plants, respectively, were tested in parallel.

### Real time PCR

To determine the expression level of the *NbRbohB* gene, leaves of *N. benthamiana* plants were infiltrated with 2.0 µM flg22 using 1 mL needleless syringe. Leaf samples were collected at 0, 0.5, 1 and 6 h post infiltration. Total RNA was isolated using the RNeasy Plant Mini Kit with DNase treatment (QIAGEN) by following the protocol provided by the manufacturer. The first-strand cDNA was synthesized using the Superscript III reverse transcriptase and oligo dT primer (Life Technologies) based on the instructions from the manufacturer. qRT-PCR was performed using gene specific primers and SYBR Green (Life Technologies) on the LightCycler^®^ 480 Instrument II (Roche), with the *NbEF1α* gene being used as an internal reference. The relative expression level of *NbRbohB* gene was calculated using the 2^−ΔΔ*CT*^ method as described [65]. The primers used for qRT-PCR are shown in the Additional file 2: Table 1.

### Transient expression of recombinant proteins in *N. benthamiana* plants

To study the suppression of programmed cell death (PCD) by AvrPtoB on the group III E2 genes-silenced (TRV-*III*) and non-silenced TRV control *N. benthamiana* plants, AvrPtoB was transiently co-expressed with the PCD elicitors AvrPto/Pto, BAX, and Avr9/Cf9, respectively using *Agrobacterium*-mediated infiltration as described [48]. Transient co-expression of the PCD elicitor and the empty pBTEX vector (EV) was used as control. The final OD_600_ of *Agrobacterium* strain GV2260 harboring *AvrPtoB* and the PCD elicitor used for transient co-expression are as the following: AvrPtoB (OD_600_ = 0.4) and AvrPto/Pto (OD_600_ = 0.4 for each); AvrPtoB (OD_600_ = 0.4) and Bax (OD_600_ = 0.4); and AvrPtoB (OD_600_ = 0.1) and Avr9/Cf9 (OD_600_ = 0.4 for each); Photographs were taken on the fourth day after infiltration.

### Electrolyte leakage assays

Cell death was assayed by measuring the electrolyte leakage from leaf discs as described previously [66, 67] with modifications. In brief, four leaf discs (6 mm in diameter) were collected from infiltrated leaf areas on the second day post infiltration (DPI) of corresponding PCD elicitor and were washed for 3 h in a 50-mL conical centrifuge tube containing 15 mL deionized pure H_2_O at room temperature (RT) with shaking at 50 rpm. The washing water in the tube was then removed and replaced with 15 mL fresh deionized pure H_2_O and the tube continued to be subjected to shaking at 50 rpm at RT. The conductivity of the bathing solution was measured using an OAKTON CON 6 conductivity meter on the third and the fourth DPI. After the measurement, the leaf discs were returned to the tube and was boiled for 25 min. After cooling down to RT, the total conductivity was measured. The percentage of conductivity measured on the third and fourth day compared to the total conductivity was calculated. For each treatment, three biological replicates were used for the assay.

## Additional files

**Additional file 1: Fig. S1.** *N. benthamiana* ubiquitin E2 enzymes are classified into thirteen groups. Numbering of the groups was based on the phylogenetic analysis of the protein sequences of the *N. benthamiana* ubiquitin E2 s and by following the numbering of the groups of tomato ubiquitin E2 s [39]. The unrooted phylogenetic tree of the amino acid sequences of the forty *N. benthamiana* ubiquitin E2s was generated by the neighbor-joining method using the MEGA6 program with 1000 bootstrap trials [61]. The Roman numerals designate the different groups.

**Additional file 2: Table** **1.** The primers used in the present research.
