# Supplementary figures and images for: Elucidating the role of highly homologous Nicotiana benthamiana ubiquitin E2 gene family members in plant immunity through an improved virus-induced gene silencing approach
Source: Plant Methods. 2017 Jul 21;13:59. doi: 10.1186/s13007-017-0210-6 (PMC5521103; doi:10.1186/s13007-017-0210-6)

## Slide 1
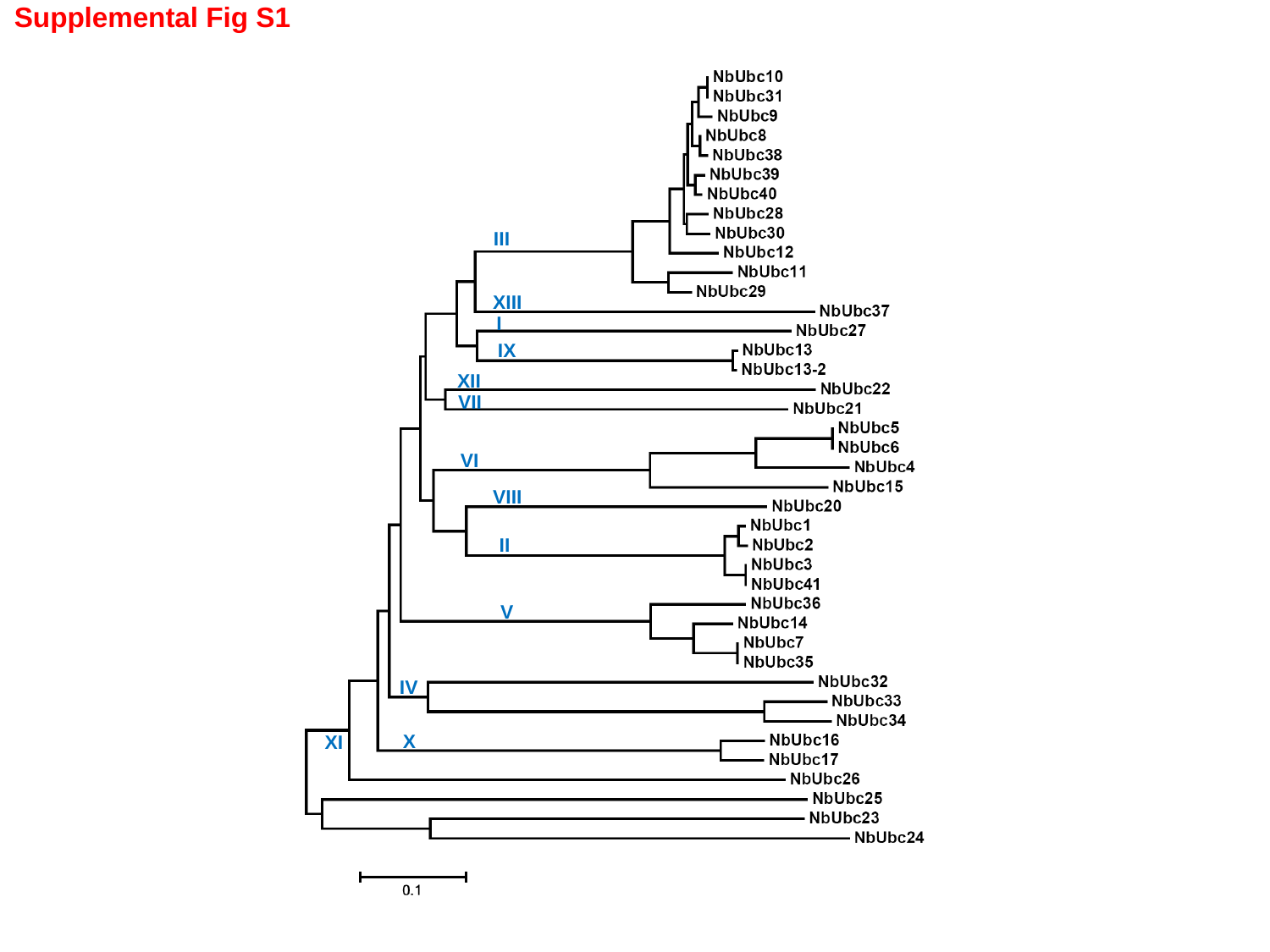

Supplemental Fig S1
III
XIII
I
IX
XII
VII
VI
VIII
II
V
IV
X
XI

Supplement: Supplementary file 1 — Additional file 1: Fig. S1. N. benthamiana ubiquitin E2 enzymes are classified into thirteen groups. Numbering of the groups was based on the phylogenetic analysis of the protein sequences of the N. benthamiana ubiquitin E2 s and by following the numbering of the groups of tomato ubiquitin E2 s [39]. The unrooted phylogenetic tree of the amino acid sequences of the forty N. benthamiana ubiquitin E2s was generated by the neighbor-joining method using the MEGA6 program with 1000 bootstrap trials [61]. The Roman numerals designate the different groups. [file 13007_2017_210_MOESM1_ESM.pptx]
