# Supplementary material for: Elucidating the role of highly homologous Nicotiana benthamiana ubiquitin E2 gene family members in plant immunity through an improved virus-induced gene silencing approach
Source: Plant Methods. 2017 Jul 21;13:59. doi: 10.1186/s13007-017-0210-6 (PMC5521103; doi:10.1186/s13007-017-0210-6)
Supplement: Supplementary file 2 — Additional file 2: Table 1. The primers used in the present research. [file 13007_2017_210_MOESM2_ESM.pdf]

Supplemental Table 1 List of primers used in this study

Name	Sequence(5'-3')	purpose
NbUBC9-GW-F	CACCATGGCATCCAAGAGGATTCT	Group III VIGS fragment *NbUBC9* cloning
NbUBC9-VIGS-R	TGTAGCCTGCCAGTGAAACAT	Group III VIGS fragment *NbUBC9* cloning
NbUBC10-VIGS-F	GAAGATATGTTTCACTGGCA	Group III VIGS fragment *NbUBC10* cloning
NbUBC10-VIGS-R	TATTTATATTTGGATGGAAAACTTTTG	Group III VIGS fragment *NbUBC10* cloning
NbUBC11-VIGS-F	CAAAAGTATTTCCATCCAAATATA	Group III VIGS fragment *NbUBC11* cloning
NbUBC11-VIGS-R	TCGTCTGGATTTGGATCTGT	Group III VIGS fragment *NbUBC11* cloning
NbUBC39-VIGS-F	ACAGATCCAAACCCAGACGA	Group III VIGS fragment *NbUBC39* cloning
NbUBC39-R	TGCTCGAGTTATCCCATCGCATATTTTTGA	Group III VIGS fragment *NbUBC39* cloning
NbUBC12-VIGS-F	ATGGGATAACTCGAGCATCTATCCATTTCC	Group III VIGS fragment *NbUBC12* cloning
NbUBC12-VIGS-R	TGCTGTTTATGTTTGGGTGG	Group III VIGS fragment *NbUBC12* cloning
NbUBC30-VIGS-F	GGTTTTCCACCCAAACATAAACAGCA	Group III VIGS fragment *NbUBC30* cloning
NbUBC30-VIGS-R	GATTAGGGTCCGTCAGAAGTG	Group III VIGS fragment *NbUBC30* cloning
Nb/slUBC12VIGS-F	CACCAGATTATCCATTCAAGCCCCC	Nonoptimized VIGS fragment cloning for silencing *NbUBC12* alone or the four E2 genes *NbUBC12* , *10* , *28* and *31.*
Nb/slUBC12VIGS-R	GCATATGGTGAAAGCAGCACCTTGGATACAGTAAG	Nonoptimized VIGS fragment cloning for silencing the three E2 genes *NbUBC10, 28* and *31* or the four E2 genes *NbUBC12* , *10* , *28* and *31.*
Nb/slUBC10VIGS-F	CACCATATGCTTGGACATACTGAAGGAGCAG	Nonoptimized VIGS fragment cloning for silencing the three E2 genes *NbUBC10* , *28* and *31* or the four E2 genes *NbUBC12* , *10* , *28* and *31* .
Nb/slUBC10VIGS-R	CACTGCAAGTACATGTGAGCAATCTCAGGAAC	Nonoptimized VIGS fragment cloning for silencing the three E2 genes *NbUBC10* , *28* and *31* or the four E2 genes *NbUBC12* , *10* , *28* and *31* .
NbUBC28VIGS-F	CACATGTACTTGCAGTGCCGGCCCCGTTGGAGAG	Nonoptimized VIGS fragment cloning for silencing the three E2 genes *NbUBC10* , *28* and *31* or the four E2 genes *NbUBC12* , *10* , *28* and *31* .
NbUBC28VIGS-R	AATGGATAGTGACTAAAAATACACCCC	Nonoptimized VIGS fragment cloning for silencing the three E2 genes *NbUBC11* , *29* , *39* and *40* .
Nb-UBC12VIGS-G-F	CACCAGTATTTGTCTGGACATCTTAAAAG	Optimized VIGS fragment cloning forsilencing *NbUBC12* alone or the four E2 genes *NbUBC12* , *10* , *28* and *31.*
Nb-UBC12VIGS-G-R	AAGCAAAACCTTGGTGAGCAGCACCTTGGATACAGT	Optimized VIGS fragment cloning forsilencing Nb-*UBC12* alone or the four E2 genes *NbUBC12* , *10* , *28* and *31.*
Nb-UBC10VIGS-G-F	CACCAAGGTTTTGCTTTCAATCTGC	Optimized VIGS fragment cloning for silencing the three E2 genes *NbUBC10, 28* and *31* or the four E2 genes *NbUBC12* , *10* , *28* and *31.*
Nb-UBC10VIGS-G-R	TTATTGTAGCTTGGTGGCAATCTCAGGAACAAGGGG	Optimized VIGS fragment cloning for silencing the three E2 genes *NbUBC10* , *28* and *31* or the four E2 genes *NbUBC12* , *10* , *28* and *31* .
Nb-UBC28VIGS-G-F	CACCAAGCTACAATAATGGGCCC	Optimized VIGS fragment cloning for silencing the three E*2 genes NbUBC10, 28 and 31 or the four E2 genes NbUBC12* , *10* , *28* and *31* .
Nb-UBC28VIGS-G-R	AATGGATAGTGACTAAAAATAC	Optimized VIGS fragment cloning for silencing the three E2 genes *NbUBC10* , *28* and *31* or the four E2 genes *NbUBC12* , *10* , *28* and *31* .
Nb-EF1a-F	AGCCTGGTATGGTTGTGACTTTTG	RT-PCR for reference gene *NbEF1a*
Nb-EF1a-R	CATGGGCTTGGTGGGAATC	RT-PCR for reference gene *NbEF1a*
Nb-UBC8-RT-F	CACCATGGCATCCAAACGGATTCTCAAAG	RT-PCR for *NbUBC8*
Nb-UBC8-RT-R	AGCCCCTCCAGAGATGGTCACT	RT-PCR for *NbUBC8*
Nb-UBC9-RT-F	CACCATGGCATCCAAGAGAATTCTGAAAG	RT-PCR for *NbUBC9*
Nb-UBC9-RT-R	CCAAATATTTGTGTTTCAGCAACTAACCC	RT-PCR for *NbUBC9*
Nb-UBC10-RT-F	CACCATGGCTTCGAAACGAATATTGAA	RT-PCR for *NbUBC10*
Nb-UBC10-RT-R	CCGCCATAGGCAATATTTAGCCCA	RT-PCR for *NbUBC10*
Nb-UBC11-RT-F	CACCATGGCATCCAGGAGAATTCA	RT-PCR for *NbUBC11*
Nb-UBC11-RT-R	CAACTCAATTCATAGCAAACTTTTGG	RT-PCR for *NbUBC11*

Nb-UBC12-RT-F	TCTTACTGTATCCAAGGTGCTGCT	RT-PCR for *NbUBC12*
Nb-UBC12-RT-R	CCAAATGTTTTCATCCCATGGCATAT	RT-PCR for *NbUBC12*
Nb-UBC28-RT-F	TAGAATTCATGGCTTCGAAACGGATATT	RT-PCR for *NbUBC28*
Nb-UBC28-RT-R	CATGGGTTAAACCGTTACCTATGG	RT-PCR for *NbUBC28*
Nb-UBC29-RT-F	CACCATGGCATCCAGGAGAATTCA	RT-PCR for *NbUBC29*
Nb-UBC29-RT-R	CTTCATGTCTTCAGACTCAGTTCATA	RT-PCR for *NbUBC29*
Nb-UBC30-RT-F	CACCATGGCTTCCAAGCGGATCT	RT-PCR for *NbUBC30*
Nb-UBC30-RT-R	TTAGCCCATGGCATACTTCT	RT-PCR for *NbUBC30*
Nb-UBC31-RT-F	CACCATGGCTTCGAAACGAATATTGAA	RT-PCR for *NbUBC31*
Nb-UBC31-RT-R	GCCGCCATAGGCAATAGTTAGCCC	RT-PCR for *NbUBC31*
Nb-UBC38-RT-F	ACGGATCCATGGCATCCAAGCGGATTC	RT-PCR for *NbUBC38*
Nb-UBC38-RT-R	CTAACCCATGGCGTACTTTT	RT-PCR for *NbUBC38*
Nb-UBC39-RT-F	AGGAATTCATGGCGTCGAAGCGCATAT	RT-PCR for *NbUBC39*
Nb-UBC39-RT-R	GGACACTTTCCGCATCATCCCATA	RT-PCR for *NbUBC39*
Nb-UBC40-RT-F	TTGAATTCATGGCGTCGAAGAGGATATT	RT-PCR for *NbUBC40*
Nb-UBC40-RT-R	TCATTCACCACAAGCACAATAAGA	RT-PCR for *NbUBC40*
Nb-UBC27-RT-F	CACCGTGGACTTGGCTAGGGTTCA	RT-PCR for *NbUBC27*
Nb-UBC27-RT-R	GCTGTGCAACAACTGCATCT	RT-PCR for *NbUBC27*
NbFni3-RT-F1	CACCAGGAATAAGTGCATCTC	RT-PCR for *NbUBC13*
NbFni3-RT-R1	ACTAGCATATAGGCGTGTCCA	RT-PCR for *NbUBC13*
